# Maize producers’ vulnerability to climate change: Evidence from Makhuduthamaga Local Municipality, South Africa

**DOI:** 10.4102/jamba.v14i1.1165

**Published:** 2022-12-07

**Authors:** Selelo Matimolane, Hector Chikoore, Fhumulani I. Mathivha, Edmore Kori

**Affiliations:** 1 Faculty of Commerce, Law and Management, University of the Witwatersrand, Johannesburg, South Africa; 2 Department of Geography and Geo-Information Sciences, University of Venda, Thohoyandou, South Africa; 3 Department of Geography and Environmental Studies, University of Limpopo, Sovenga, South Africa; 4 Department of Hydrology, University of Zululand, Kwadlangezwa, South Africa

**Keywords:** adaptation, climate change, maize yields, rain days, rainfall, temperature, trends, vulnerability

## Abstract

Climate change is predicted to impact agricultural production and affect food security in poor communities of developing countries due to the likely negative impacts on rainfall characteristics. South Africa is one of the largest producers of maize crops in the Southern African Development Community (SADC) region. The majority of crop production is rainfed with precipitation received during the summer growing season. This study evaluated the impact of climate change on maize yields using trend and multiple regression analysis in northern South Africa. Exposure and vulnerability of maize farmers to the impacts of climate change were also evaluated. Rainfall characteristics showed variability of 20.35% with rain days standard deviation of 10.25 days and coefficient of variation of 18.57%. The results revealed a weak relationship between annual rainfall and rainy days, and annual rainfall and maize yields, both showed an *r*² and *p*-values of less than 0.5 and 0.005, respectively. The study found that variations in rainfall did not significantly influence variation in maize yields. Despite a clear fluctuation in yields, the results demonstrate a rising trend that can be attributed to agricultural practices such as the use of fertilisers and planting drought resistant cultivars as opposed to climate variables. The study further found that maize producers were proactively adapting to climate change, thus, reducing their vulnerability to its impacts.

## Introduction

Agricultural production depends on climatic conditions to produce high yields, thereby rendering the agriculture sector vulnerable to the impacts of changing climatic conditions. The contribution and importance of agricultural production to the economies of various countries differ. Countries with a large portion of the economy in agriculture face a larger exposure to the impacts of climate change than countries with a lower share of agricultural contribution to their economy (IPCC 2014). Southern African economies are sensitive to the direct impacts of climate change due to their dependence on rainfed agriculture. This is further exacerbated by high poverty levels and geographic exposure (Moeletsi & Walker 2012) including unreliable and highly variable rainfall. Understanding the influence of climate variability and change on agricultural production is essential to cope with projected changes in temperatures and precipitation patterns. Maize is the most widely produced crop (FAO 2018), largest locally produced (Ambrosino, Chandler & Todd 2014) and an important grain crop (Moeletsi, Mphethe & Tsubo 2016) in South Africa. It is both a major component of livestock feed and the staple food for the majority of the South African population. Moeletsi et al. (2013) indicated that the climate of Limpopo Province is often characterised by extreme weather events such as intense rainfall, droughts and heat waves. While the maize crop is produced under diverse environments (Du Plessis 2003), for the case of South Africa three major growing regions, western region (35%), eastern region (45%) and Kwa-Zulu Natal region (10%) are produced under rainfed conditions (Haarhoff, Kotzé & Swanepoel 2020). Zhao et al. (2017) suggests that a sustained rise in temperature and altered rates of precipitation are most likely to have a negative impact on crop yields. Blignaut et al. (2009) further reported that a 1% rainfall decline can lead to a 1.1% decline in maize yields during the summer season.

In 2017, South Africa’s Department of Agriculture, Forestry and Fisheries reported that from 2010, Limpopo Province received below-normal to near-normal rainfall, while temperatures were on average above normal. High temperatures and inadequate rainfall during the critical stages of growth adversely affect crop production (Raza et al. 2019). Rural communities of Limpopo Province are dependent on maize and vegetable farming for their livelihood (Musetha 2016). Erratic and extreme climatic conditions are expected to impact the quantity of yields achieved (Mpandeli et al. 2005). According to Tshiala et al. (2011), the changing climatic conditions severely burden rural communities in Limpopo Province. According to a 2019 Intergovernmental Panel on Climate Change (IPCC) report, under the climate change scenarios that project a hotter and drier climate, food security, access, price increases, and market pricing stability will be affected by changing climatic conditions. Producers dependent on rainfall continue to face numerous risks associated with agricultural production (Imbach et al. 2017). Climate change is expected to disproportionately affect smallholder farmers and make their livelihoods even more precarious (Harvey et al. 2014). This is because farmers are frequently exposed to pests, disease outbreaks, and extreme weather events which cause significant crop and income losses. Temperature is an important environmental factor affecting insect population dynamics, and as such, global climate warming could trigger an expansion of their geographic range, increased risk of invasive insect species and insect-transmitted plant diseases (Skendžić et al. 2021). The World Bank (2016) projects that unless the world acts to fight climate change, 100 million more people could be driven into poverty by the year 2030. Maize production in South Africa usually exhibits variations in yield, closely related to fluctuations in seasonal rainfall (Goldblatt 2010; Haarhoff et al. 2020). The Bureau for Food and Agricultural Policy (2016) reports that South Africa’s maize production reached 11.8 million tons during 2013–2014 and consisted of 5.6 million tons of white maize and 6.2 million tons of yellow maize. The subsequent 2014–2015 season yielded crops of 14.2 million tons. Although a significant increase in yield is noted between the two seasons, the demand to meet the needs of the country’s growing population and export demands may not be sufficiently met. Accordingly, the variation is of concern because local consumption of maize has increased in line with a growing population and the existing export trade demands. The increase in demand for maize crops will affect both local and regional supply.

Maize production and storage are vulnerable to climate change (Lacambra et al. 2020), and this vulnerability is also propagated to maize producers especially in developing countries such as South Africa. This can be attributed to a low level of technological advancement, lack of resources to mitigate the negative consequence of climate change, and variability in agricultural production (Nath & Behera 2011). This provides a strong motivation to examine how varying climatic change scenarios affect crop productions and yields. Several studies have reported on the probable effects of climate variability and change on rainfed agricultural systems in southern Africa (Kanyepi & Tanyanyiwa 2016; Nhemachena et al. 2020; Olabanji, Ndarana & Davis 2021). However, the impacts on maize crop yields and how they vary over time have received less attention (Osborne & Wheeler 2013). Therefore, this study focuses on the impacts of climate change on maize yields and further assesses maize producers’ vulnerability to these changes in Makhuduthamaga Local Municipality, northern South Africa.

## Methodology

### Description of the study area

**FIGURE 1:**
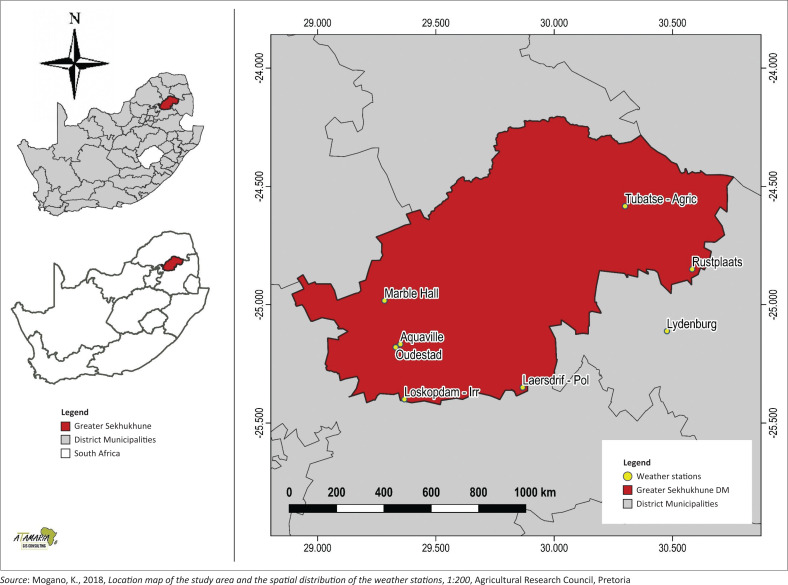
Location map of the study area and the spatial distribution of the weather stations in the study area.

The study was conducted in the Makhuduthamaga Local Municipality situated within the Sekhukhune District Municipality of Limpopo Province, northern South Africa (Figure 1). The municipality covers an area of 2 097 km² and is located in the northern parts of South Africa. The number of households within the municipal area increased from 49 797 households in 1996 to a total of 65 217 in 2011 with a population of 274 358 according to the 2011 census (Statistics South Africa 2012). Certain areas are considered highly suitable for agriculture with an adequate supply of water for irrigation and receive over 80% of its total rainfall between September and March, at times extending to April. The average annual rainfall ranges from 500 mm to 800 mm with the mean average annual temperature of 20 °C (Petja, Nesamvuni & Nkoana 2014). The area has a mean average growing season of about 167 days. The high evaporation risk results in low moisture supply capacity. Irrigation is therefore essential for cultivated farming practices (Petja et al. 2014).

### Maize, rainfall and temperature data

Rainfall and temperature data for the period under study (1985–2015) for nine weather stations were obtained from the South African Weather Service. Stations with uninterrupted data at > 90% up-to-date records were selected for the analysis. The World Meteorological Organization (WMO) recommends a period of 30 years or longer as ideal for studies dealing with long-term changes (WMO 2017). Data on maize production and yields for the period under study was obtained from the South African National Department of Agriculture.

### Data analyses

The standard deviations and sample variance were used to analyse and demonstrate maize yields variation against the total growing area and rainfall for the study area. The descriptive statistical analysis was conducted using Microsoft excel and cross tabulation.

### Impacts of climate varability on maize production

Multiple regression was utilised to establish the impact of climate variability on maize production. This test of relation was summarised with the *p*-value. The significance threshold of the *p*-value was set at < 0.05. To decide whether this relationship is positive or negative, the correlation coefficient was determined. The R^2^ value varies between –1.0 to 1.0, where –1.0 shows a negative significant trend while 1.0 indicated a positive significant trend.

### Determining producers’ vulnerability to climate change

The vulnerability assessment method (VAM) (Schröter & Metzger 2004) was used to determine maize producers’ vulnerability. The method is based on a conceptual function describing how the different elements of vulnerability are related to each other. The three components of vulnerability as defined by the IPCC (2014) are sensitivity, exposure, and adaptive capacity. For this study, a total of 76 questionnaires were distributed to the maize producers during focus group discussions at the Makhuduthamaga Local Municipality. Six focus group sessions were conducted with each group made up of between 10 and 14 producers. The focus group participants were selected using purposive and convenience sampling methods. With all administered questionnaires completed, the study had a response rate of 100%. The questionnaire was developed and used to solicit biographical and socio-economic information (age, gender, educational level), producers’ perceptions, and vulnerability to climate variability and change. Data on access to agricultural extension support, the impact of climate variability and change on maize production were also obtained through the questionnaire. A five point Likert scoring system (where 1 is very low, 2 is low, 3 is medium, 4 is high, and 5 is extreme) described in Fontaine and Steinman (2009) was used to assess exposure, sensitivity, and adaptive capacity of each maize producer sampled. The Likert scale ranking corresponds to a score as shown in Table 1. Thus, vulnerability is a function of exposure, sensitivity, and adaptive capacity as shown in Equation 1 (e.g. Fontaine & Steinman 2009; Metzger & Schröter 2006).

$$V=\frac{E+S}{A}$$ [Eqn 1]

where, *V* is Vulnerability, *E* is Exposure; *S* is Sensitivity, and *A* is Adaptive capacity.

**TABLE 1:** Vulnerability component scoring system.

Ranking scale	Score
**Exposure**	
Extreme	5
High	4
Moderate	3
Low	2
Very low	1
**Sensitivity**	
Extreme	5
High	4
Moderate	3
Low	2
Very low	1
**Adaptive capacity**	
Extreme	5
High	4
Moderate	3
Low	2
Very low	1

*Source*: Fontaine, M.M. & Steinemann A.C., 2009, ‘Assessing vulnerability to natural hazards: Impact-based method and application to drought in Washington State’, *Natural Hazard Review* 10(1), 11–18

### Ethical considerations

To protect the respondents’ privacy, the researchers obtained informed consent from them to participate voluntarily in the study. They were assured that their information would not be misused to embarrass or humiliate them. Only data absolutely necessary for achieving the objectives of the study would be obtained. To promote awareness of ethical principles and issues in conducting research activities while using human participants in the research study, ethical clearance was sought and obtained from the University of Venda Reseach Ethics Committee, reference number: SES/18/GGIS/12/0709.

## Results

### Rainfall characteristics in Makhuduthamaga Local Municipality

Table 2 shows rainfall characteristics and maize yield statistics for the study area for the period 1985–2015. The highest number of rainy days in the rainfall season observed during the study period were 76 days, and this was observed in 1996–1997 while the lowest number of 34 rainy days were observed in 2003–2004, which was a drought season. The highest and lowest rainy days observed showed a significant positive relationship (*r*² > 0.5 and *p*-value < 0.01) with annual rainfall. Thus, the number of rainfall days has the greatest influence on the total rainfall received in the study area over the study period. These findings agree with those by Ojo, Ojo and Oni (2001), which showed that the patterns of mean rainy days generally follow the pattern with mean rainfall amounts. However, the strength of this relationship might weaken as the number of heavy rainfall days increases due to climate change, such that most rainfall would fall in fewer rainy days. The rainy days’ standard deviation of 10.25 days and the coefficient of variance of 18.57% characterises the annual occurrences of rainy days as consistent.

**TABLE 2a:** Rainfall characteristics and maize yield in the study area.

Year	Maize yields (ton/hec.)	Total annual rainfall (mm)	Rainfall days	Rainfall onset	Rainfall cessation
1985	N/A	587.4	57	28-Sept	15-Mar
1986	1.8	557.2	55	05-Oct	01-Apr
1987	1.3	697.6	70	30-Sept	27-Mar
1988	2.17	554.5	63	03-Oct	22-Mar
1989	1.62	644.6	66	27-Sept	29-Mar
1990	2.65	620.5	59	23-Sept	02-Apr
1991	0.61	645.3	61	28-Oct	05-Apr
1992	1.29	414.5	39	07-Oct	16-Mar
1993	2.09	628.1	62	25-Sept	01-Apr
1994	1.18	505.4	51	01-Oct	22-Mar
1995	3.25	541.7	55	29-Sept	28-Mar
1996	2.25	802.1	76	17-Sept	11-Apr
1997	2.45	510.5	49	04-Oct	24-Mar
1998	1.47	449.9	45	02-Oct	18-Mar
1999	3.01	438.2	44	30-Sept	20-Mar
2000	2.22	752.4	71	20-Sept	05-Apr
2001	2.5	544.0	57	27-Sept	22-Mar
2002	2.72	435.4	46	01-Oct	17-Mar
2003	2.88	266.2	34	26-Oct	03-Mar
2004	2.76	531.8	49	03-Oct	10-Mar
2005	3.5	362.5	37	11-Oct	17-Mar
2006	2.4	617.9	58	30-Sept	23-Mar
2007	4	443.3	40	08-Oct	11-Mar
2008	5.2	689.3	66	24-Sept	06-Apr
2009	5	579.4	60	28-Sept	30-Mar
2010	5	558.1	61	01-Oct	27-Mar
2011	4.8	561.3	55	03-Oct	22-Mar
2012	5.5	602.6	58	26-Sept	19-Mar
2013	6.1	646.9	63	25-Sept	07-Apr
2014	7.7	535.5	55	02-Oct	26-Mar
2015	5.8	484.4	49	05-Oct	28-Mar

*Source:* Author’s computation from data obtained from the South African Weather Services 2018, South Africa’s meteorological data files 1985–2015

**TABLE 2b:** Rainfall characteristics and maize yield in the study area.

Variable	Maize yields (ton/hec.)	Total annual rainfall (mm)	Rainfall days
Mean	3.174	555.1	55.194
Standard deviation	1.729	113.0	10.248
Coefficinet of variation (%)	54.47	20.35	18.57

*Source:* Author’s computation from data obtained from the South African Weather Services 2018, South Africa’s meteorological data files 1985–2015

Rainfall onset and cessation in the study area occurs during late September to early October and March to early April, respectively. Studies such as Tadross et al. (2007) and Tshililo, Savage and Moeletsi (2021) defined rainfall season onset in the summer rainfall region of South Africa as the 1st October; however, for this study, false onset was also considered similar to Mathivha (2020) and as such rainfall received in late September was included in the analysis. About 49% of the rainfall seasons received the first rains in late September while only 1996 received the first rains in mid September (17 September 1996). The other half of the years studied received the first rains in early October which coincides with the onset in summer rainfall regions of the country. The date of occurrence of either the rainfall onset or cessation plays a vital role in defining the start and end of the growing season (Moeletsi, Mellaart & Mpandeli 2011). Olanrewaju (2006) identified rainfall onset and cessation as important components of moisture resource status for determining the potential of various crops in Lagos, Nigeria. Similarly, Adamge and Ujoh (2013) reported a strong correlation between maize yields and rainy season characteristics in Nigeria. Therefore, aligning planning dates to rainfall onset is a key factor in producing high crop yields under rainfed conditions (Ati, Stigter & Oladipo 2002; Raes et al. 2004). Maize producers benefit from early rains by planting at the earliest rainfall onset during the wet season while during the dry years, producers use early maturing maize varieties that are drought resistant. Statistical analysis of rainfall characteristics in the study area indicates that rainfall is highly variable at 20.35%, and thus poses a risk to maize production. Studies have shown that high coefficients of variation are not uncommon in semi-arid environments. A coefficient of variation of annual rainfall of up to 26% has been reported in South Africa’s Limpopo Province and North West Province, respectively (Kosgei 2008; Lynch et al. 2001).

### Rainfall variation and trends during maize growing seasons

Table 3 shows the annual variation of rainfall over the main and the secondary growing seasons in the study area. The average annual rainfall was found to be 555.1 mm with lowest rainfall totals of 266.2 mm and 362.5 mm observed in 2003 and 2005, respectively. Annual rainfall totals exceeding 600 mm were recorded in 10 different years over the period of study, wherein the highest was 802.1 mm in 1996. Dependent on the distribution of rainfall over the rainy season, years with rainfall in access of 450 mm per annum would indicate that the water requirements for the life cycle of a maize crop were met without negatively affecting production. Du Plessis (2003) reported that when annual rainfall totals fall below 450 mm per annum, maize crops are more likely to be negatively affected due to water deficits while Moeletsi and Walker (2012) further supported this by indicating that areas receiving less than 400 mm of rainfall in dry years are vulnerable to soil water deficits.

**TABLE 3:** Variation of annual rainfall over the growing season.

Stations	Mean of the main growing season	Mean of the second growing season	Standard deviation		Coefficient of variation	
			Main growing season	Second growing season	Main growing season (%)	Second growing season (%)
Mantrombi	382.1	128.8	22.1	28.1	5.8	21.8
Marble hall	361.5	110.8	26.0	29.4	7.2	26.5
Loskop Dam – IRR	377.2	98.0	30.6	24.9	8.1	25.4
Laersdrif – Police	428.5	112.6	31.1	29.0	7.3	25.7
Lydenburg	303.0	106.7	11.7	22.9	3.8	21.5
Tubatse Agric	250.3	89.3	18.8	20.8	7.5	23.3
Rustplaats	358.2	128.1	29.6	24.8	8.3	19.4
Aquaville	379.0	101.4	24.6	22.7	6.5	22.3
Oudestad	311.1	98.9	18.7	25.1	6.0	25.3

*Source:* Author’s computation from data obtained from the South African Weather Services 2018, South Africa’s meteorological data files 1985–2015

The descriptive statistics show that for the main growing season (October–January), mean rainfall is higher than during the second growing season in all the weather stations. The results showed that rainfall during the growing seasons was highly variable. For the main growing season, the station which received the most rainfall is Laersdrif – Police recording a mean seasonal rainfall of 645.5 mm. Tubatse Agric station was the driest with mean rainfall of 250.3 mm, a 50% variation in rainfall. During the secondary growing season, results show that Mantrombi station was the most wet, with a mean seasonal rainfall of 128.8 mm, while Tubatse Agric is the driest (with 89.3 mm). Distinct variations in annual rainfall can be identified in all weather stations; the secondary growing seasons’ Coefficinet of Variation (CV) are higher than that of the main growing season. For the main growing season, Rustplaats weather station had the highest CV of 8.3% while Lydenburg weather station had the lowest CV of 3.8%. Variability existed in temporal inter-seasonal rainfall in all the weather stations, ranging from 250.3 mm to 428.5 mm for the main growing season and 371 mm – 601 mm for the secondary growing season.

### Temperature variation and trends during maize growing seasons

**FIGURE 2:**
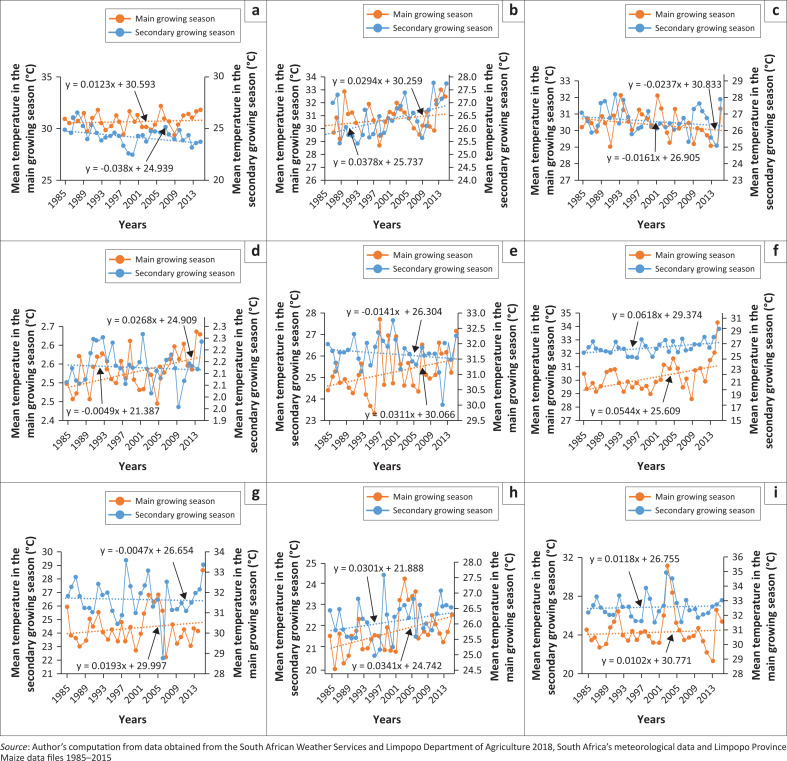
Mean temperature trends during maize main and secondary growing seasons for; (a) Aquaville, (b) Mantrombi, (c) Laesdrif-Police, (d) Tubatse-Agric station, (e) Rustplaats, (f) Oudestad, (g) Loskop Dam, (h) Lydenburg and (i) Marble Hall.

Figures 2 (a–i) show the variation and trends of temperature during the maize growing season. In the main growing season, eight weather stations showed an upward trend with the exception of Laersdrif weather station, which showed a statistically insignificant trend of –0.0161 °C per year for the main growing season and –0.0237 °C for the secondary growing season. Oudestad weather station recorded the greatest increase in the maximum temperature for both growing seasons, 0.062 °C and 0.054 °C per year for the main growing season and secondary growing season, respectively. The study findings further revealed that variance in temperature has increased over the study period, with *r*² > 0.5. Thus, the changes in temperature are becoming more predictable. The average maximum temperatures were higher between 2006 and 2015 (at 26.8 °C) than in the preceding decade (1995–2004) with average temperatures at 26.5 °C. Tshiala et al. (2011) reported an increase of 0.12 °C in the mean annual temperature per decade, between 1950 and 1999 while Kruger and Nxumalo (2017) noted a decrease in wet spells over north-eastern South Africa. Furthermore, Strzepek et al. (2011) projected that in southern Africa climate change will result in the rise in temperature, evaporative demands, and changes in rainfall and runoff patterns. The findings on increased temperature over the study period and changes in rainfall and runoff patterns have the potential to threaten maize production in the study area. Maitah, Malec and Maitah (2021) reported a decrease in maize yield in the Czech Republic after the year 2010 and this was attributed to decreased precipitation and an increase in temperature.

### Maize yields variability

The standard deviations and sample variance were used to demonstrate maize yields variation for the study area. Overall, the study showed significant variability in maize yields both spatially and temporally, with *r*² of greater than 0.5. Maize yields have continually varied and are closely linked with the climate variables. Despite a clear fluctuation in yields, the results demonstrate a clear upward trend that may not be accounted for by climate variation but to other farming factors such as increased area planted or the use of drought resistant cultivars. The effect of the total area grown and variations in rainfall tend to influence maize yields. The most unproductive year by yields per hectare planted over the study period was in the 1991–1992 growing season with total rainfall of 414.5 mm received over 39 days in that year. The 1991–1992 summer was characterised by one of the most severe droughts over southern Africa (Chikoore & Jury 2021). The most productive season was 2014–2015 with a production rate of 7.7 tons per hectare. This success may be attributed to the total rainfall received of 535.5 mm in that growing season. For the period under study, the variations observed in the average rainfall and rain days were not related to the variation in the yield of maize. The results revealed a weak relationship between annual rainfall, rain days, and maize yields (*r*² > 0.5, *p* > 0.05, and *r*² > 0.5, *p* > 0.05, respectively). On further analysis of the rainfall data, it was concluded that these results are skewed by several major outliers noted in 1996, 2003, and 2005. Maize yields outliers were identified in 2013, 2014, and 2015. These were constrained to provide unbiased results. The results showed a positive but insignificant relationship between annual rainfall and rain days and annual rainfall and maize yields with both R² and *p* values less than 0.5 and 0.05, respectively.

### Producers’ vulnerability to climate change

Vulnerability assessment (VA) indicates that producers are highly exposed to climate variability and change. Department of Environmental Affairs (2013) reported that for the period 2080–2100, temperatures over Limpopo Province are projected to increase, reaching a remain never observed before in the region. At the regional level, Kruger and Shongwe (2004), Engelbrecht et al. (2015), and Kruger and Nxumalo (2017) reported a strong and dangerous warming trend over southern Africa. Furthermore, agricultural extension services are meant to provide producers with appropriate information on a variety of issues regarding crop production such as alternative methods, technologies, adaptation strategies, and technical skills. At a global and regional scale, extreme weather events have the potential to increase the risk of multiple simultaneous crop failure (Mehrabi & Ramankutty 2019; Tigchelaar et al. 2018) and this may also be the case for producers in the study area as there have been reports of increased extreme events in the region. Vulnerability is a function of exposure, sensitivity, and adaptive capacity (Fontaine & Steinman 2009; Metzger & Schroter 2006), and their average values were used in computing the overall producers’ vulnerability. Exposure was scored the highest at 0.8, the sensitivity of producers was 0.74. Adaptive capacity was low with a value of 0.38 with a vulnerability of 4.05. Thus, based on the vulnerability indicator scoring system, producers have high vulnerability to climate variability and change. Studies show that climate change exposes rural-based subsistence farmers to new and unfamiliar conditions (Leichenko & O’Brien 2000). Erratic rainfall patterns and increasing temperatures without adaptation strategies pose a risk to agricultural production. This is expected to intensify the producer’s vulnerability to climate variability and change (Atedhor 2016). While producers are not passive participants in climate change adaptation and actively adapt, others continue to face increased vulnerability, particularly in the developing world such as sub-Saharan Africa (Chagutah 2010; Thornton et al. 2006).

### Adaptive capacity and producer’s adaptation measures to climate change and variability

Table 4 shows climate variability and change adaptive capacity indicators used by maize farmers. The VA results show that supplemental irrigation scored the highest adaptive indicator among the producers while intercropping is the lowest indicator. The low value for moderate adaptive capacity finding agrees with the findings by Maponya and Mpandeli (2012) who found that only 21% of producers have implemented an adaptation measure to mitigate the perceived impacts of climate variability and change. Tshiala et al. (2011) suggested that producers in Limpopo Province might be able to adapt to increased temperatures and rainfall variability; however, the frequency of extreme weather events may have negative impacts on the producers’ community.

**TABLE 4:** Farmer’s adaptive capacity indicators to climate change and variability.

Indictor	Score	Description
Supplemental irrigation	0.65	High adaptive capacity
Planning planting seasons based on the first rains	0.40	Moderate adaptive capacity
Intercropping	0.20	Low adaptive capacity
Production inputs	0.38	Moderate adaptive capacity
Crop rotation	0.30	Moderate adaptive capacity
Drought resistant crop varieties	0.40	Moderate adaptive capacity
Adaptive capacity	0.38	Moderate adaptive capacity

Table 5 shows the adoption ranking of adaptation measures to climate variability and change. The results of the cross-tabulation showed that 33% of female producers and 59% of male producers opted for the use of supplemental irrigation. Between both genders, the age group 46–60 was the most active across all age groups. Only 21% of female producers adopted crop rotation while 28% of males adopted the same measure. Maddison (2007) and Anley, Bogale and Haile-Gabriel (2007) indicated that improving employment is key to increasing the adoption of various adaptation measures. About 77% of the farmers were most active across all adopted measures identified, both in terms of uptake and non-uptake of adaptation measures. Producers’ uptake of adaptation measures indicates that many have opted for more than one adaptation measure. The adoption ranking results show that supplemental irrigation ranked 92% as the preferred method of adaptation. The use of production inputs was ranked second while crop rotation was ranked the lowest. Up to 83% of producers acknowledged the absence of external support, as they relied on farming experience, available finances, and knowledge of their environment to select an adaptation measure deemed suitable to the existing weather condition.

**TABLE 5:** Adoption ranking of adaptation measure to climate variability and change.

Adaptation measure	Number of producers	Ranking	Percentages
Supplemental irrigation	70	1	92.11
Production inputs	61	2	80.26
Intercropping	57	3	75.00
Planning planting seasons based on the first rains	45	4	59.21
Drought resistant crop varieties	40	5	52.63
Crop rotation	37	6	48.68

## Discussions

Seasonal rainfall and maximum temperatures showed significant variation in the historical record over the study area. As expected, the main growing season received higher rainfall compared to the secondary growing season. However, the results also showed higher rainfall variability during the main growing season than the secondary growing season, with a general negative trend across several stations. Maximum temperatures have shown an upward increase of about 0.02 °C since 1985. Over the main growing season, eight out of the nine weather stations showed an upward trend and warming in maximum temperatures, and only Laersdrif station recorded a decline during both growing seasons. The Oudestad weather station recorded the highest increase in maximum temperatures for both growing seasons. Maize yields revealed substantial variations in both spatial and temporal terms. High inter-annual and intra-seasonal rainfall variability over most parts of southern Africa affects agricultural productivity (Moeletsi et al. 2013). Over the study period, maize yields have constantly varied and are closely associated with climate variability. However, despite the fluctuations in maize yields due to climate change, the long-term trend is upward suggesting other scientific and technological interventions such as improved crop varieties (Chikoore & Jury 2021). Akpalu, Hassan and Ringler (2009) determined that precipitation is an important driver of maize production. Thus, from the study results, it is evident that the variations in climatic variables tend to have a significant impact on maize yields. The unpredictability in CV values reveals the impacts of climatic variables on maize, with variability in maize yields related to the main growing season rainfall. Exposure, sensitivity and adaptive capacity to climate variability and change render producers highly vulnerable. Furthermore, erratic rainfall patterns and increasing temperatures without supplemental irrigation pose a threat to producers’ ability to produce sustainably and avoid crop failure and reduced yields. The changing climatic conditions are expected to exacerbate producers’ agricultural vulnerability to climate variability and change (Atedhor 2016). Producers’ vulnerability to climate change can be minimised through the timely adoption of adaptation measures (Schubert et al. 2007). As such, the potential for agricultural adaptation at the farm level is very high because adaptations arise from the farmers’ perception of changed or changing conditions. In the study, 61.5% of the producers have implemented or adopted an adaptation strategy to cope with the perceived climate variability and change. Uddin, Bokelmann and Entsminger (2014) found that farmers’ responses to the effects of climate change and adoption of adaptation strategies are influenced by their socio-economic characteristics, with knowledge of the farmers being the most influential. The results also demonstrate that most producers have adopted more than one adaptation measure. Several studies (i.e. Hou, Huang & Wang 2015; Kom et al. 2019) have shown that the effects of climate change will be felt by small rural farmers due to their lack of adequately adaptive capacity as they depend on rainfed farming. The study showed some relationship between the adoption of adaptation measures to address climate variability and change and independent variables such as age and farming experience.

## Conclusion

The scientific community has broadly acknowledged that climate variability and change phenomena are an occurring reality. This will undeniably have negative impacts on developing nations whose economies are dependent on agriculture. The study determined maize producer’s vulnerability and assessed the impacts of climate variability and change on maize production in the Makhuduthamaga Local Municipality. The study further identified adaptation measures adopted by producers in response to climate change and variability. Mean temperatures are on an increasing trend and this was shown by 89% of the weather stations. These increasing trends and rainfall distribution over the study area are anticipated to increase the occurrence and magnitude of extreme climatic events such as floods and droughts. Extreme events have been found to affect maize production because the amount and timing of rainfall is an important driver of maize production. The effect of variations of temperature and rainfall on maize yields is influenced by the trend magnitude and direction of each of the climatic variables. These changes pose a risk to food security and livelihoods in the rural communities of Limpopo Province. Producers are proactively adapting to climate change and thus reducing their vulnerability to the impacts of climate change. Agricultural extension plays an important role in encouraging farmers to adopt new technologies in place of traditional methods, improve skills among others. Access to agricultural support and improved methods and seed technology appears to be one of the major challenges in the subsistence farming sector. In this study, it was found that demographic household profiles such as age, farming experience, and occupation were important determinants in the adoption of adaptation measures.

## References

[CIT0001] Adamge, M.E. & Ujoh, F., 2013, ‘Effect of variability in rainfall characterisation on maize yield in Gboko, Nigeria’, *Journal of Environmental Protection* 4(9), 881–887. 10.4236/jep.2013.49103

[CIT0002] Akpalu, W., Hassan, R.M. & Ringler, C., 2009, *Climate variability and maize yield in South Africa: Results from GME and MELE methods*, International Food Policy Research Institute Discussion Paper No. 843, International Food Policy Research Institute, Washington, DC.

[CIT0003] Ambrosino, C., Chandler, R.E. & Todd, M.C., 2014, ‘Rainfall-derived growing season characteristics for agricultural impacts assessment in South Africa’, *Theoretical and Applied Climatology* 115, 411–426. 10.1007/s00704-013-0896-y

[CIT0004] Anley, Y., Bogale, A. & Haile-Gabriel, A., 2007, ‘Adoption decision and use intensity of soil and water conservation measures by smallholder subsistence farmers in Dedo district, Western Ethiopia’, *Land Degradation and Development* 18(3), 289–302. 10.1002/ldr.775

[CIT0005] Atedhor, G., 2016, ‘Changing pattern of rainfall peak in the sudano-sahelian region of Nigeria’, *International Journal Renewable Energy & Environment* 2, 211–221.

[CIT0006] Ati, O., Stigter, C.J. & Oladipo, E., 2002, ‘A comparison of methods to determine the onset of the growing season in Northern Nigeria’, *International Journal of Climatology* 22(6), 731–742. 10.1002/joc.712

[CIT0007] Blignaut, J., Ueckermann, L. & Aronson, J., 2009, ‘Agriculture production’s sensitivity to changes in climate in South Africa’, *South African Journal of Science* 105(1–2), 61–68.

[CIT0008] Bureau for Food and Agricultural Policy (BFAP), 2016, *Agricultural outlook 2016–2025*, viewed 13 November 2017, from http://www.bfap.co.za/documents/baselines/BFAP_Baseline_2016.pdf.

[CIT0009] Chagutah, T., 2010, *Climate change vulnerability and preparedness in Southern Africa: Zimbabwe Country Report*, Heinrich Boell Stiftung, Cape Town.

[CIT0010] Chikoore, H. & Jury, M.R., 2021, ‘South African drought, deconstructed’, *Weather and Climate Extremes* 33, 100334. 10.1016/j.wace.2021.100334

[CIT0011] Department of Environmental Affairs, 2013, *Long term adaptation scenarios flagship research programme (LTAS) for South Africa*, Climate Trends and Scenarios for South Africa, Department of Environmental Affairs, Pretoria.

[CIT0012] Du Plessis, J., 2003, *Maize production*, Department of Agriculture, Directorate Agricultural Information Services Private Bag X144, Pretoria, p. 38.

[CIT0013] Engelbrecht, F., Adegoke, J., Bopape, M.J., Naidoo, M., Garland, R., Thatcher, M. et al., 2015, ‘Projections of rapidly rising surface temperatures over Africa under low mitigation’, *Environmental Research Letters* 10(8), 085004. 10.1088/1748-9326/10/8/085004.

[CIT0014] Fontaine, M.M. & Steinemann, A.C., 2009, ‘Assessing vulnerability to natural hazards: Impact-based method and application to drought in Washington State’, *Natural Hazard Review* 10(1), 11–18. 10.1061/(ASCE)1527-6988(2009)10:1(11)

[CIT0015] Food and Agricultural Organisation (FAO), 2018, *FAOSTAT database*, FAO, Rome, viewed 19 February 2018, from http://www.fao.org/faostat/en/#data.

[CIT0016] Goldblatt, A., 2010, *Agriculture: Facts & trends, South Africa*, World Wide Fund for Nature, Cape Town, viewed 19 February 2018, from http://awsassets.wwf.org.za/downloads/facts_brochure_mockup_04_b.pdf.

[CIT0017] Haarhoff, S.J., Kotzé, T.N. & Swanepoel, P.A., 2020, ‘A prospectus for sustainability of rainfed maize production systems in South Africa’, *Crop Science* 60(1), 14–28. 10.1002/csc2.20103

[CIT0018] Harvey, C.A., Lalaina Z.R., Nalini, S., Radhika, D., Razafimahatratra, H., Hasinandrianina, R. et al., 2014, ‘Extreme vulnerability of smallholder farmers to agricultural risks and climate change in Madagascar’, *Philosophical Transactions of the Royal Society B* 369(1639), 20130089. 10.1098/rstb.2013.0089 PMC3928894 24535397

[CIT0019] Hou, L.L., Huang, J.K. & Wang, J.X., 2015, ‘Farmers’ perceptions of climate change in China: The influence of social networks and farm assets’, *Climate Research* 63(3), 191–201. 10.3354/cr01295

[CIT0020] Imbach, P., Beardsley, M., Bouroncle, C., Medellin, C., Läderach, P., Hidalgo, H. et al., 2017, ‘Climate change, ecosystems and smallholder agriculture in Central America: An introduction to 51 the special issue’, *Climatic Change* 141(1), 1–12. 10.1007/s10584-017-1920-5

[CIT0021] Intergovernmental Panel on Climate Change (IPCC), 2014, ‘Climate change 2013: The physical science basis’, In press *Contribution of Working Group I to the Fifth Assessment Report of the Intergovernmental Panel on Climate*, Cambridge University Press, Cambridge.

[CIT0022] Intergovernmental Panel on Climate Change (IPCC), 2019, ‘Food security’, in P.R. Shukla, J. Skea, E. Calvo Buendia, V. Masson-Delmotte, H.-O. Pörtner, D.C. Roberts et al. (eds.), *Climate change and land: An IPCC special report on climate change, desertification, land degradation, sustainable land management, food security, and greenhouse gas fluxes in terrestrial ecosystems*, IPCC, Geneva.

[CIT0023] Kanyepi, T. & Tanyanyiwa, V.I., 2016, ‘Impacts of climate change on rain-fed agriculture in Matope Ward, Mt Darwin District, Zimbabwe’, *International Journal of Development and Sustainability* 5(4), 187–198.

[CIT0024] Kom, Z., Nethengwe, N.S., Mpandeli, S. & Chikoore, H., 2019, ‘Climate change grounded on empirical evidence as compared with the perceptions of smallholder farmers in Vhembe District, South Africa’, *Journal of Asian and African Studies* 55(5), 683–698. 10.1177/0021909619891757

[CIT0025] Kosgei, J.R., 2008, ‘Rainwater harvesting systems and their influences on field scale soil hydraulic properties, water fluxes and crop production’, Ph.D. thesis, University of KwaZulu-Natal, Pietermaritzburg.

[CIT0026] Kruger, A. & Shongwe, S., 2004, ‘Temperature trends in South Africa: 1960–2003’, *International Journal of Climatology* 24(15), 1929–1945. 10.1002/joc.1096

[CIT0027] Kruger, A.C. & Nxumalo, M.P., 2017, ‘Historical rainfall trends in South Africa: 1921–2015’, *Water SA* 43(2), 285. 10.4314/wsa.v43i2.12

[CIT0028] Lacambra, C., Molloy, D., Lacambra, J., Leroux, I.,Klossner, L., Talari, M. et al., 2020, *Factsheet resilience solution for the maize sector in South Africa*, Inter-American Development Bank, Washington, DC.

[CIT0029] Leichenko, R.M. & O’Brien, K.L., 2000, ‘The dynamics of rural vulnerability to global change: The case of southern Africa’, *Mitigation and Adaptation Strategies for Global Change* 7, 1–18.

[CIT0030] Lynch, S.D., Zulu, J.T., King, K.N. & Knoesen, D.M., 2001, ‘The analysis of 74 years of rainfall recorded by the Irwins on two farms south of Potchefstroom’, *Water SA* 27(4), 559–564. 10.4314/wsa.v27i4.4970

[CIT0031] Maddison, D., 2007, *The perception of and adaptation to climate change in Africa*, Policy Research Working Paper 4308, World Bank, Washington DC.

[CIT0032] Mogano, K., 2018, *Location map of the study area and the spatial distribution of the weather stations, 1:200*, Agricultural Research Council, Pretoria.

[CIT0033] Maitah, M., Malec, K. & Maitah, K., 2021, ‘Influence of precipitation and temperature on maize production in the Czech Republic from 2002 to 2019’, *Scientific Reports* 11, 10467. 10.1038/s41598-021-89962-2 34001991 PMC8129148

[CIT0034] Maponya, P. & Mpandeli, S., 2012, ‘Climate change and agricultural production in South Africa: Impacts and adaptation options’, *Journal of Agricultural Science* 4(10), 48. 10.5539/jas.v4n10p48

[CIT0035] Mathivha, F.I., 2020, ‘Drought in Luvuvhu River Catchment – South Africa: Assessment, characterisation, and prediction’, Unpublished PhD thesis submitted to the University of Venda, Thohoyandou.

[CIT0036] Mehrabi, Z. & Ramankutty, N., 2019, ‘Synchronized failure of global crop production’, *Nature Ecology & Evolution* 3, 780–786. 10.1038/s41559-019-0862-x 30988491

[CIT0037] Metzger, M.J. & Schröter, D., 2006, ‘Towards a spatially explicit and quantitative vulnerability assessment of environmental change in Europe’, *Regional Environmental Change* 6, 201–216. 10.1007/s10113-006-0020-2

[CIT0038] Moeletsi, M.E., Mellaart, E.A.R. & Mpandeli, N.S., 2011, ‘Crop water requirements analysis for maize trial sites in Makhado during 2007/08 season’, in S.D. Attri, L.S. Rathore, M.V.K. Sivakumar & S.K. Dash (eds.), *Challenges and opportunities in agrometeorology*, pp. 485–490, Springer, Berlin, Heidelberg.

[CIT0039] Moeletsi, M.E., Mellaart E.A.R., Mpandeli, N.S. & Hamandawana, H., 2013, ‘The use of rainfall forecasts as a decision guide for small-scale farming in Limpopo Province, South Africa’, *The Journal of Agricultural Education and Extension* 19(2), 133–145. 10.1080/1389224X.2012.734253

[CIT0040] Moeletsi, M.E., Mphethe, T. & Tsubo, M., 2016, ‘The study of frost occurrence in Free State Province of South Africa’, *Advances in Meteorology* 2016, 1–9. 10.1155/2016/9586150

[CIT0041] Moeletsi, M.E. & Walker, S., 2012, ‘Rainy season characteristics of the Free State Province of South Africa’, *Water SA* 38(5), 775–782. 10.4314/wsa.v38i5.17

[CIT0042] Mpandeli, S., Simalenga, T., Siambi, M., Ramgondo, R., Mailula, N. & Liphadzi, K., 2005, *Constraints and challenges to agricultural development in Limpopo Province, South Africa*, 2nd International Forum on Water and Food, CGIAR Challenge Program on Water & Food, Addis Ababa, Ethiopia, November 10–14, 2008.

[CIT0043] Musetha, M.A., 2016, *The impact of climate change on agricultural crop production in the Vhembe District Municipality, Limpopo Province South Africa*, University of South Africa, Pretoria, viewed 18 February 2018, from http://hdl.handle.net/10500/22068.

[CIT0044] Nath, P.K. & Behera, B., 2011, ‘A critical review of impact and adaptation to climate change in developed and developing countries’, *Environmental Development Sustainability* 13, 141–162. 10.1007/s10668-010-9253-9

[CIT0045] Nhemachena, C., Nhamo, L., Matchaya, G., Nhemachena, C.R., Muchara, B., Karuaihe, S.T. et al., 2020, ‘Climate change impacts on water and agriculture sectors in Southern Africa: Threats and opportunities for sustainable development’, *Water* 12(10), 2673. 10.3390/w12102673

[CIT0046] Ojo, O., Ojo, K. & Oni, F., 2001, *Fundamentals of physical and dynamic climatology*, SESEC Publishers, Lagos.

[CIT0047] Olabanji, M.F., Ndarana, T. & Davis, N., 2021, ‘Impact of climate change on crop production and potential adaptive measures in the Olifants Catchment, South Africa’, *Climate* 9(1), 6. 10.3390/cli9010006

[CIT0048] Olanrewaju, R.M., 2006, ‘Implication of late onset of rains in a Coastal Ecological Area: The case study of Lagos and its environs’, *Geo-Studies Forum* 3(1 & 2), 83.

[CIT0049] Osborne, T.M. & Wheeler, T.R., 2013, ‘Evidence for a climate signal in trends of global crop yield variability over the past 50 years’, *Environmental Research Letters* 8(2), 024001. 10.1088/1748-9326/8/2/024001

[CIT0050] Petja, B.M., Nesamvuni, E. & Nkoana, A., 2014, ‘Using geospatial information technology for rural agricultural development planning in the Nebo Plateau, South Africa’, *Journal of Agricultural Science* 6(4), 10–18. 10.5539/jas.v6n4p10

[CIT0051] Raes, D., Sithole, A., Makarau, A. & Millford, J., 2004, ‘Evaluation of first planting dates recommended by criteria currently used in Zimbabwe’, *Agricultural and Forest Meteorology* 125(3–4), 177–185. 10.1016/j.agrformet.2004.05.001

[CIT0052] Raza, A., Razzaq, A., Mehmood, S.S., Zou, X., Zhang, X., Lv, Y. et al., 2019, ‘Impact of climate change on crops adaptation and strategies to tackle its outcome: A review’, *Plants* 8(2), 34. 10.3390/plants8020034 30704089 PMC6409995

[CIT0053] Schröter, D. & Metzger, M.J., 2004, ‘Global change vulnerability – Assessing the European human-environment system’, *Proceedings, Twentieth Sessions of the Subsidiary Bodies (SB 20), United Nations Framework Convention on Climate Change (UNFCCC), Workshop on Scientific, Technical, and Socio-Economic Aspects of Impacts of Vulnerability and Adaptation to Climate Change*, viewed 21 February 2018, from http://unfccc.int/files/meetings/workshops/other_meetings/application/pdf/schroeter.pdf.

[CIT0054] Schubert, R., Schellnhuber, H.J., Buchmann, N., Epiney, A., GrieBhammer, R., Kulessa, M. et al., 2007, *Climate change and security risk*, Earthson, London.

[CIT0055] Skendžić, S., Zovko, M., Pajač Živković, I., Lešić, V. & Lemić, D., 2021, ‘Effect of climate change on introduced and native agricultural invasive insect pests in Europe’, *Insects* 12(11), 985. 10.3390/insects12110985 34821786 PMC8619401

[CIT0056] Statistics South Africa, 2012, *Census 2011 Statistical release – P0301.4*, Statistics South Africa, Pretoria.

[CIT0057] Strzepek, K., McCluskey, A., Boehlert, B., Jacobsen, M. & Fant, I., 2011, *Climate variability and change: A Basin scale indicator approach to understanding the risk to water resources development and management*, Water Papers, The World Bank, Washington, DC.

[CIT0058] Tadross, M., Suarez, P., Lotsch, A., Hachigonta, S., Mdoka, M., Unganai, L. et al., 2007, ‘Changes in growing-season rainfall characteristics and downscaled scenarios of change over southern Africa: Implications for growing maize’, in *IPCC regional expert meeting on regional impacts, adaptation, vulnerability, and mitigation*, Nadi, Fiji, pp. 193–204.

[CIT0059] Thornton, P.K., Jones, P.G., Owiyo, T.M., Kruska, R.L., Herrero, M., Kristjanson, P. et al., 2006, *Mapping climate vulnerability and poverty in Africa*, Report to the Department for International Development, The International Livestock Research Institute (ILRI), Nairobi.

[CIT0060] Tigchelaar, M., Battisti, D.S., Naylor, R.L. & Ray, D.K., 2018, ‘Future warming increases probability of globally synchronized maize production shocks’, *Proceedings of the National Academy of Sciences USA* 115(26), 6644–6649. 10.1073/pnas.1718031115 PMC6042138 29891651

[CIT0061] Tshiala, M.F., Olwoch, J. & Engelbrecht, A.F., 2011, ‘Analysis of temperature trends over Limpopo Province, South Africa’, *Journal of Geography and Geology* 3(1), 13. 10.5539/jgg.v3n1p13

[CIT0062] Tshililo, F.P., Savage, M.J. & Moeletsi, M.E., 2021, ‘Rainy season characteristics for the Luvuvhu River catchment, South Africa’, *Water SA* 47(4), 480–487.

[CIT0063] Uddin, M.N., Bokelmann, W. & Entsminger, J.S., 2014, ‘Factors affecting farmers’ adaptation strategies to environmental degradation and climate change effects: A farm level study in Bangladesh’, *Climate* 2(4), 223–241. 10.3390/cli2040223

[CIT0064] World Bank; IFC; MIGA, 2016, *World Bank Group Climate Change Action Plan 2016–2020*. World Bank, Washington, DC.

[CIT0065] World Meteorological Organization (WMO), 2017, *WMO guidelines on the calculation of climate normal*, WMO-No. 1203, Geneva.

[CIT0066] Zhao, C., Liu, B., Piao, S., Wang, X., Lobell, D.B.,Juang, Y. et al., 2017, ‘Temperature increase reduces global yields of major crops in four independent estimates’, *Proceedings of the National Academy of Sciences USA* 114(35), 9326–9331. 10.1073/pnas.1701762114 PMC5584412 28811375

